# Helmet Radio Frequency Phased Array Applicators Enhance Thermal Magnetic Resonance of Brain Tumors

**DOI:** 10.3390/bioengineering11070733

**Published:** 2024-07-19

**Authors:** Faezeh Rahimi, Bilguun Nurzed, Thomas W. Eigentler, Mostafa Berangi, Eva Oberacker, Andre Kuehne, Pirus Ghadjar, Jason M. Millward, Rolf Schuhmann, Thoralf Niendorf

**Affiliations:** 1 Berlin Ultrahigh Field Facility, Max-Delbrück-Center for Molecular Medicine in the Helmholtz Association, 13125 Berlin, Germany; bilguun.nurzed@mdc-berlin.de (B.N.); mostafa.berangi@mdc-berlin.de (M.B.); eva.oberacker@mdc-berlin.de (E.O.); jason.millward@mdc-berlin.de (J.M.M.); 2 FG Theoretische Elektrotechnik, Technical University of Berlin, 10587 Berlin, Germany; rolf.schuhmann@tu-berlin.de; 3 Technische Universität Berlin, Chair of Medical Engineering, 10587 Berlin, Germany; thomas.eigentler@mdc-berlin.de; 4 Berliner Hochschule für Technik, 13353 Berlin, Germany; 5 MRI.TOOLS GmbH, 13125 Berlin, Germany; andre.kuehne@gmail.com; 6 Department Radiation Oncology, Charité–Universitätsmedizin Berlin, Corporate Member of Freie Universität Berlin and Humboldt-Universität zu Berlin, Augustenburger Platz 1, 13353 Berlin, Germany; pirus.ghadjar@charite.de; 7 Experimental and Clinical Research Center, Joint Cooperation between Charité Unversitätsmedizin and the Max-Delbrück Center for Molecular Medicine in the Helmholtz Association, 13125 Berlin, Germany

**Keywords:** brain tumor, glioblastoma multiforme, theranostics, temperature, hyperthermia, MRI, Thermal Magnetic Resonance, RF transmitter array, helmet RF phased array, multi-target evaluation approach

## Abstract

Thermal Magnetic Resonance (ThermalMR) integrates Magnetic Resonance Imaging (MRI) diagnostics and targeted radio-frequency (RF) heating in a single theranostic device. The requirements for MRI (magnetic field) and targeted RF heating (electric field) govern the design of ThermalMR applicators. We hypothesize that helmet RF applicators (HPA) improve the efficacy of ThermalMR of brain tumors versus an annular phased RF array (APA). An HPA was designed using eight broadband self-grounded bow-tie (SGBT) antennae plus two SGBTs placed on top of the head. An APA of 10 equally spaced SGBTs was used as a reference. Electromagnetic field (EMF) simulations were performed for a test object (phantom) and a human head model. For a clinical scenario, the head model was modified with a tumor volume obtained from a patient with glioblastoma multiforme. To assess performance, we introduced multi-target evaluation (MTE) to ensure whole-brain slice accessibility. We implemented time multiplexed vector field shaping to optimize RF excitation. Our EMF and temperature simulations demonstrate that the HPA improves performance criteria critical to MRI and enhances targeted RF and temperature focusing versus the APA. Our findings are a foundation for the experimental implementation and application of a HPA en route to ThermalMR of brain tumors.

## 1. Introduction

Localized hyperthermia (HT, temperature range T = 39–44 °C for 60–90 min) is used as a thermal adjunct therapy that has been proven to enhance chemo-, radio- or immunotherapy to inhibit tumor growth and improve the efficacy of the clinical outcome [1,2]. Thermal therapy has seen a revival for the treatment of glioblastoma multiforme (GBM), which is the most frequent and aggressive malignant brain tumor, comprising more than 60% of all brain tumors [3] and with a mean survival time of approximately 14 to 15 months from diagnosis [4]. Targeted radiofrequency (RF)-induced heating is a specific variant of thermal therapy [2,5] considered for glioblastoma therapy. Notwithstanding the clinical benefits, standalone HT approaches lack inherent in vivo diagnostic imaging, non-invasive temperature mapping, and therapy detection capabilities [1,6,7]. Any in vivo HT modality would greatly benefit from diagnostic guidance, temperature dosimetry, and the capacity to monitor therapy outcomes, which is facilitated by non-invasive imaging [8,9]. This is especially crucial for GBM thermal therapy planning and monitoring because of the constraints provided by the skull, and the very sensitive nature of the central nervous system [10].

Magnetic resonance imaging (MRI) is a mainstay of diagnostic imaging. It offers exquisite spatial resolution and soft tissue contrast for anatomical reference. MRI provides intrinsic contrast mechanisms for functional and physiological assessment. Moreover, MRI facilitates non-invasive temperature cartography to support HT therapy planning and monitoring [11,12,13,14]. This makes it conceptually appealing to synergistically connect MRI and HT in a multi-modality approach. Current MRI-HT hybrid implementations commonly employ two RF sub-systems that are controlled and operated independently [15,16]. This approach requires extra RF hardware and is not cost-effective. The risk of mutual interferences between the two RF chains is a weakness of the multi-modality hybrid approach. This approach may induce compatibility issues and practical obstacles for the system’s integration because each sub-device is typically provided by different vendors.

An instrument that truly integrates diagnostic guidance, thermal treatment, and therapy monitoring can help better define the role of temperature in biological systems and disease and use these insights for enhanced thermal therapies. Thermal Magnetic Resonance (ThermalMR) adds a thermal intervention dimension to a MRI device. It integrates RF-induced heating, in vivo non-invasive temperature mapping, and diagnostic MRI in a single, multi-purpose RF applicator including a phased array of RF transmitters [17]. ThermalMR is facilitated by the two orthogonal and complementary components of electromagnetic fields. RF transmission and reception used for MRI rely on magnetic fields (H-field) perpendicular to the main static magnetic field (B_0_) of a MR scanner. RF heating builds on electric fields (E-field). Constructive interference of E-fields produced by multiple RF sources can be used for targeted focal RF heating. These requirements govern the design of multi-purpose RF applicators tailored for ThermalMR.

MR instruments operating at ultrahigh magnetic field strengths (UHF, B_0_ ≥ 7.0 T) [18] use higher operating frequencies than conventional clinical MRI systems. While the long electromagnetic wavelength in tissue at 64 MHz (1.5 T, commonly used for clinical MRI) is not suitable to focus electromagnetic (EM) energy within small target volumes, the shortened wavelength in tissue at ≥297 MHz (≥7.0 T) enables stronger focusing of RF fields due to more localized interference patterns. This phenomenon offers the potential to provide focal temperature increase, offering ample opportunity for ThermalMR theranostics.

Ensuring a patient and case-specific adaptation of the size, uniformity, and location of the RF energy deposition in the treatment target volume is of the essence for developing ThermalMR theranostics for brain tumors [10,19,20,21]. The focal point quality is governed by the radiation pattern of the single RF transmit element [22,23,24,25,26], the thermal intervention frequency of the RF applicator, the RF channel count, and the geometric arrangement of the transmit elements in a phased array. The potential of high-density phased arrays for ThermalMR RF applicators is well recognized [10,17,19,25,27,28]. Increasing the number of RF transmission elements per unit area increases the degrees of freedom (amplitude and phase) and enhances the focal point quality and RF peak power deposition for thermal therapy. It also enables improvements in the transmission field (B_1_^+^) efficiency and uniformity of MRI. Considering the limited amount and costs of RF amplifiers of commercially available MRI systems which typically feature 8TX channels, distributing a limited number of RF transmission elements around the head presents an alternative to increasing the number of RF transmission elements.

Pioneering reports on the ultimate intrinsic signal-to-noise ratio (SNR) in MRI and ultimate intrinsic specific absorption rate (SAR) suggested that RF transmit antenna arrangement and positioning also influences MR image quality in UHF-MR, as well as the focal point quality of thermal interventions [29,30]. This is especially relevant for ThermalMR of deep-seated brain tumors. The head presents a small surface area that constrains the number of RF transmission elements that can be arranged in an array to cover the head. Multi-channel transmit RF arrays tailored for whole brain MRI or HT of the head are commonly designed with loop or dipole antennas [10,21,28,31], or combinations of both loop and dipole antennas [22,32,33,34]. Dome-shaped helmet RF applicator configurations provide a viable alternative for MRI or HT [21,25,31,35,36,37]. However, the dome-shaped RF arrays previously published did not incorporate transmission elements positioned perpendicular to either the cranial–caudal axis of the body or the main magnetic field of an MR scanner. The applicability and performance of this approach for ThermalMR have not yet been explored. The conventional antenna phased arrays (APAs) utilized in current applications feature a singular row of elements. Our research diverges from this standard by not simply adding more elements or rows to increase complexity. Instead, we present a strategy that retains the simplicity inherent in most MRI-guided hyperthermia (MRI-HT) applicators. We achieve this by reconfiguring the APA into a helmet-like array, offering a novel approach that differs significantly from methods discussed in the existing literature. This allows for enhanced functionality without complicating the array structure, aligning with our goal of improving treatment efficacy while maintaining device simplicity.

Recognizing this opportunity, we hypothesize that dome-shaped helmet RF applicators improve the efficacy of ThermalMR theranostics by providing enhanced mean B_1_^+^ uniformity for brain MRI at 7.0 T and by enabling enhanced focal point quality for RF-induced heating of brain tumors versus an annular RF array using the same number of transmit RF elements. To test this hypothesis, this work examines the feasibility and benefits of a helmet ThermalMR applicator comprising eight broadband self-grounded bow-tie (SGBT) antennae [24,38,39] arranged in an annular phased array around the head in conjunction with two SGBT antennae placed on top of the head perpendicular to B_0_. For benchmarking, an annular ThermalMR applicator comprised of 10 equidistantly placed transmit SGBT elements is used as a reference. To assess the ThermalMR applicators, we first used the conventional approach of targeting a few very specific but arbitrary areas of the brain. Recognizing that this approach does not represent the full clinical picture we expanded the evaluation to whole-brain slice coverage. For this purpose, we introduce a multi-target evaluation (MTE) framework.

## 2. Materials and Methods

### 2.1. RF Applicator Design

#### 2.1.1. MRI Considerations

**Figure 1:**
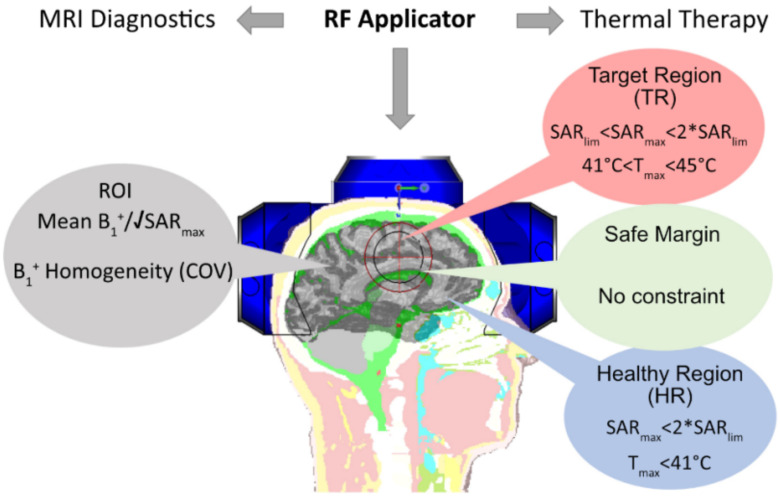
ThermalMR integrates MRI diagnostics and thermal therapy in a single RF applicator. MRI diagnostics and thermal therapy have different requirements and quality metrics in the region of interest (ROI), healthy region (HR), target region (TR), and safety margin [10]. RF transmission field efficiency and uniformity are critical to diagnostic MRI. Targeted RF power deposition governs the focal point quality of RF-induced thermal therapy.

For diagnostic MRI, transmission field (B_1_^+^, B_1_^+^ = (B_x_ + iB_y_)/2 efficiency, and B_1_^+^ homogeneity are critical criteria for RF array design (Figure 1) [40]. RF elements used for MRI should induce an H-field (H~ B_1_^+^) perpendicular to the main static magnetic field B_0,z_. Considering that only B_xy_ components contribute to B_1_^+^, appropriate RF element arrangement is critical for MRI. Using a multi-channel array of RF elements enhances B_1_^+^ uniformity in the target region while staying within the SAR (W/kg) limits of MRI [40,41,42].

#### 2.1.2. RF Heating Considerations

RF heating builds on E-fields and depends on the extent of energy absorption in tissue, which is described by the SAR or Power Loss Density (PLD) in W/m^3^. Thermal dosage is the integral of SAR over time [43]. In HT, a SAR-induced temperature increase is used to heat the target region between T = 39 and 44 °C while preserving surrounding healthy tissues (T < 41 °C) and maintaining a safe margin [44] between the target region and healthy tissue (Figure 1).

These requirements for MRI and RF heating govern the design of RF applicators tailored for ThermalMR.

#### 2.1.3. RF Antenna Building Block

**Figure 2:**
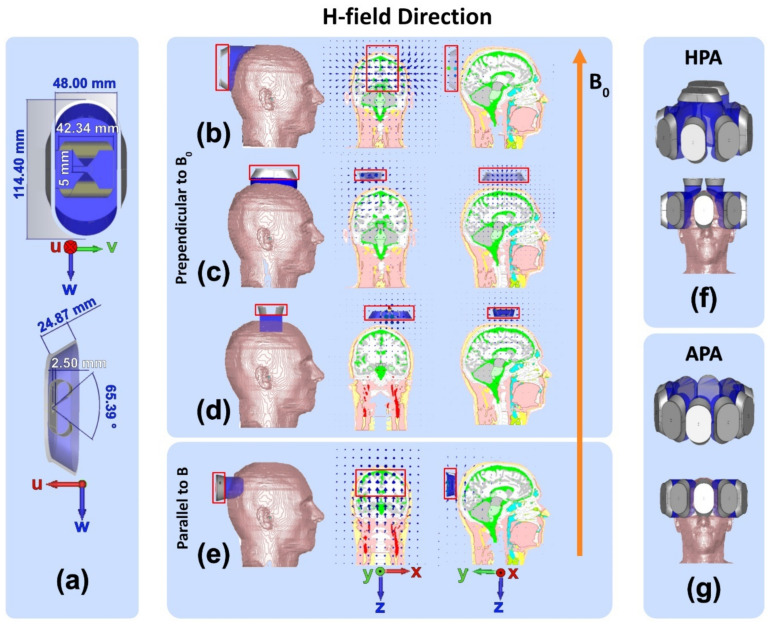
Broadband self-grounded bow-tie (SGBT) antenna and H-field distributions in the human voxel model Duke. (**a**) Front and side view of a SGBT antenna in the uvw-coordinate system. H-field distributions are shown for the head of the human voxel model Duke using an xyz-coordinate system for (**b**) H-field induced by a SGBT antenna (specified by a red border) with the long axis being aligned along the superior-inferior direction (*y*-axis) of the head; (**c**,**d**) H-field induced by a SGBT antenna placed on top of the head with the long axis being aligned along the y (**c**) or the x-direction (**d**) of the head model. (**e**) Dark MRI mode H-field induced by a SGBT antenna placed perpendicular to B_0_ with the long axis aligned along the x-direction of the head model. The static magnetic field B_0_ is in the z-direction. Schematic views of the ThermalMR applicators using a (**f**) helmet and (**g**) annular array comprising ten SGBT building blocks.

A broadband SGBT antenna [45] (Figure 2a, frequency range: 230–560 MHz, size: 42.3 × 46.3 × 20 mm^3^, antenna: 0.3 mm copper, substrate: 0.5 mm FR4) was used as an RF building block for modeling transmission (TX) of electromagnetic waves [24]. For wavelength shortening, the SGBT was placed in a housing (Nylon 12, 114.4 × 54 × 22.5 mm^3^) filled with D_2_O (ε = 81 at 297.2 MHz to reduce antenna size) [24]. D_2_O was used because the MRI resonance frequency is outside of the bandwidth of the SGBT antenna. Hence, the SGBT building block does not contribute to the MR signal, which H_2_O would otherwise do. In this work, the xyz-coordinate system describes the orientation of the MR scanner and of the object under investigation (OUI). The uvw-coordinate system describes the orientation of the SGBT which is shown in Figure 2. Considering the defined uvw-coordinate system and microscopic Maxwell’s equations for propagating electromagnetic plane waves, SGBTs generate an E-field (V/m) in the ±w-axis direction and an H-field (A/m) perpendicular to the E-field (S = E × H, where S is the Poynting vector in the direction of propagation [46]) and the propagation direction in the far-field (Fraunhofer Distance) of antenna (>2 D^2/^$\lambda_{c},$ when D (√(42.3^2^ × 46.3^2^ × 20^2^) = 65.8 mm) is the maximum dimension of the antenna profile) [24,47,48]. At 297.2 MHz, the wavelength inside the D_2_O-filled antenna building block is λ_c_ = 113.9 mm, so the far-field starts at distances >76.7 mm. Figure 2 shows the H-field vector pattern of an SGBT building block for four arrangements on the human head. Configurations shown in Figure 2b–d provide H-fields perpendicular to B_0_. For example, an SGBT antenna placed perpendicular to B_0_ (w-axis parallel to *z*-axis) with the long axis aligned with the anterior-posterior (*y*-axis) direction generates an H-field perpendicular to B_0_ (Figure 2b). When placing the SGBT on top of the head with the vw-plane parallel to the xy-plane and the w-axis parallel to the *y*-axis (Figure 2c) or the w-axis parallel to the *x*-axis (Figure 2d), H-fields are generated in the x-direction and y-direction, which are perpendicular to B_0_. The configuration with the w-axis of the SGBT aligned with the y-direction (Figure 2c) provides better coverage along the long axis of the head than the configuration, where the w-axis of the SGBT is aligned with the *x*-axis (Figure 2d). Therefore, the former was used for further examination. If the SGBT antenna is rotated 90° around its u-axis and aligned with the v-axis parallel to the *z*-axis, the induced H-fields are produced in the z-direction parallel to B_0_ (Figure 2e). The latter setup represents the “dark MRI mode” and is excluded from further considerations since it does not provide an H-field perpendicular to B_0_ [49].

#### 2.1.4. RF Phased Array

For the design of the RF applicator array, the inter-element distance was considered first in order to prevent creating grating lobes (GLs). GLs have much higher intensity than side lobes, which can lead to severe power losses. The RF antenna array’s radiation pattern can be calculated through [50,51]:

(1) $$\theta_{GL}=\sin^{-1}\left(sin\theta_{0}\pm \frac{2n\pi \lambda_{c}}{d}\right),n=0,\;1,\;2,\;3,\ldots ,$$

where *n* is the number of grating lobes (*n* = 0 is the main lobe), and *d* is the inter-element distance. According to Equation (1), the inter-element distance should be $\frac{\lambda_{c}}{4}<d<\lambda_{c}$. Considering the inter-element distance requirements and the size of an average human head, two RF applicators, each comprising ten RF building blocks (Figure 2), were designed:

- **Annular Phased RF array** (APA, Figure 2g): Ten SGBT RF building blocks were placed equidistantly in a single-row annular array where the w-axis of the SGBT elements is aligned with the *z*-axis of the main magnetic field B_0_.
- **Helmet Phased RF array** (HPA, Figure 2f): Eight SGBT RF building blocks shown in Figure 2b were arranged equidistantly in a single-row annular array. Two SGBT RF building blocks, shown in Figure 2c, were placed on top of an eight-elements APA. This approach takes the elliptical shape of the human head into account and aligns the long axis of the SGBT with the longest axis of the head for the benefit of better head/brain coverage.

The shortest inter-element distance between the feeding points of adjacent RF building blocks is 83 mm for the APA and 105 mm for the annular ring of the HPA. A water bolus filled with deionized water (H_2_O) (T = 21 °C) was placed between the RF building blocks and the OUI for antenna matching and surface cooling.

### 2.2. Numerical Simulations

#### 2.2.1. EMF Simulations

For the design and the assessment of the two RF applicator configurations, EMF simulations were performed using two commercial software packages:

- **CST Microwave Studio Suite 2020** (Dassault Systèmes, Darmstadt, Germany) [10,52]: EMF simulations were performed using broadband excitation (f = 297.2 ± 50 MHz) and the time-domain solver based on the finite integration technique (FIT) mesh size of 1.75 mm × 1.75 mm × 1.75 mm was used for the antenna. EMF simulations were applied to the human head voxel model Duke of the virtual family truncated at the level of the neck (IT’IS Foundation, Zurich, Switzerland [53]) (resolution of 1.0 × 1.0 × 1.0 mm^3^) and placed at the isocenter of a RF shield model of the bore of a 7.0 T MRI system. For the human voxel model, SAR_10g_ (SAR calculations were averaged over 10 g of tissue or phantom material (SAR_10g_)) was evaluated for cuboid target regions (TR). For this purpose, four TR sizes with a main target region (mm^3^) and a gap between the target margin and safety margin (mm^3^) were defined: TR_1_ = 87.5 mm × 87.5 mm × 4 mm (22 mm), TR_2_ = 62.5 mm × 62.5 mm × 4 mm (10 mm); TR_3_ = 37.5 mm × 37.5 mm × 4 mm (10 mm); TR_4_ = 15 mm × 15 mm × 4 mm (10 mm). The cuboid TRs were chosen over cylindrical or spherical TRs to make the TR more tumor shaped, unpredictable, with some corners, independent from the symmetric applicator arrangement of a circular array, and more challenging than a cylindrical TR. To examine the homogeneity of SAR_10g_, the metric target coverage (TC) describes the target volume that covers xx% (xx = 25, 50, and 75) of the maximum SAR_10g_ (SAR_10g,max_) inside the TR.
- **Sim4Life Version 7.0.2** (Zurich Med Tech, Zurich, Switzerland). The Electromagnetics Full Wave Solvers finite-difference time-domain (P-EM-FDTD) was used for EM modeling (f = 297.2 ± 50 MHz). Thermodynamic Solvers (P-THERMAL) based on FDTD and a steady-state finite volume were used for advanced perfusion and thermoregulation modeling. The P-Thermal solver utilizes the Poisson differential equation to model heat transfer in living tissue, accommodating a range of adaptable boundary conditions. The transient thermal solver assumes a dynamic state where all tissue domains possess non-zero thermal conductivity or heat transfer rates. This software package supports the import of segmented real-world data obtained from computed tomography (CT) or MRI into a human voxel model. It also provides a comprehensive library of thermal and electrical tissue properties for a human model. For the simulations, clinical tumor geometry data obtained from a GBM patient were integrated into the human voxel model truncated at the level of the neck. For this purpose, a CT scan of a GBM patient was imported into Sim4Life [10]. Dielectric and thermal properties of up to 20 labeled tissue [10] used for radiotherapy planning including the GBM (volume = 172 mL, σ = 1.15 S/m, ε = 66.5 [54]) were assigned to the head geometry of the patient (headmass = 3.68 kg). Adding the cerebrospinal fluid (CSF) layer was accomplished by upscaling the brain by 5% [10]. The excitation center frequency and bandwidth were set to 297.2 ± 50 MHz. The mesh size, regarding the voxel shaping of the antenna in Sim4Life was limited to a maximum step size of 3 mm within the skull and 5 mm within the lower region of the modified human head voxel model. A much finer resolution of down to 0.1 mm was applied to the feeding points to resolve the triangular shape of the SGBT.

#### 2.2.2. Temperature Simulations

Temperature simulations were performed with Sim4Life using the Pennes bioheat transfer equation including tissue perfusion with a thermal simulation time of t = 3600 s [55,56].

In the Pennes bioheat transfer equation heat exchange mechanisms, including heat conduction, blood perfusion, and resistive heating are considered, as follows [57]:

(2) $$C(r)\;\rho (r)\frac{\partial T(r,t)}{\;\partial t}=\nabla \cdot (K(r)\nabla T(r,t))+\rho (r)SAR(r)+A(r,t)-B(r,t)(T(r,t)-TB(r,t))$$

where the position vectors of tissue and time are denoted by r and t, respectively, and the temperatures of tissue and blood are *T* and *TB* (°C), respectively. *C* (J/°C/kg) is the specific heat of tissue, *K* (W/°C/m) is the thermal conductivity of the tissue, *ρ* (kg/m^3^) is mass density; and *A* (r, t) is the heat generated by metabolism. The initial temperature of the water bolus was fixed at *T* = 21 °C. The initial body temperature was set to *T* = 37 °C [58]. Subsequently, the transient heating phase was evaluated for an intervention period of 3600 s using the thermal transient solver considering the thermal boundaries of the European Society for Hyperthermic Oncology (ESHO) guidelines [54]. To simulate the effect of RF heating, the temperature was calculated from the previously calculated power loss density W/cm^3^ (local SAR_10g_) based on the Pennes bioheat equation. Temperature-dependent thermal properties using the thermal stress model based on the ESHO benchmarks were assigned to the patient head voxel model. The blood perfusion rate under thermal stress is different for fat, muscle, tumor, and skin considering the tumor is typically inhomogenous, well perfused in the periphery, and necrotic in the core (due to lack of blood perfusion). For instance, this value increases from 0.7 to 3.6 in muscle [54]. For the HT optimizer tool of Sim4Life [59], clinical TV (CTV) and gross TV (GTV) were chosen as target tissues. The loss-free power received in the tumor was defined as 11 W per RF channel inside the tumor to reach a maximum of about 45 °C in the tumor. Cumulative histograms of 10%, 50%, and 90% of the masked target region (here CTV) covered by a minimum temperature are given by T10, T50, and T90 (°C).

### 2.3. RF Circuit Co-Simulation

While a SGBT exhibits a broadband frequency response, its implementation in a phased array design with multiple antennas in APA and HPA introduces coupling effects that can impact its frequency response. By adding a lossy L-series and a C-parallel circuit in Matlab 2021a (Mathworks, Natick, MA, USA), co-simulations for frequency tuning and impedance matching of each of the 10-element RF arrays were performed to ensure S_ii_ (return loss) and S_ij_ (mutual coupling) of <−10 dB for all RF elements [50]. The evaluation of estimated losses involved analyzing the equivalent series resistance of capacitors with data sourced from non-magnetic ceramic capacitors (ATC100C, American Technical Ceramics, NY, USA). Inductor losses were taken into consideration by using the Q-factor, referring to the database for non-magnetic air-coil inductors (1512SP, Coilcraft Inc., Cary, IL, USA). Subsequently, the results from electromagnetic field simulations, coupled with material/tissue properties, were utilized in post-processing (Matlab) to calculate distributions of B_1_^+^ and SAR_10g,max_, achieving an isotropic resolution of 4.0 × 4.0 × 4.0 mm^3^. The −10 dB bandwidth (Δf|_Sii<−10_) is calculated for both phased arrays.

### 2.4. Electromagnetic Field Shaping

#### 2.4.1. Transmission Field Shimming for MRI

To enhance B_1_^+^ transmission efficiency and uniformity over the entire brain and to manage RF safety, transmission field shimming was performed. For this purpose, a genetic optimization algorithm [60] was used in conjunction with unconstrained minimization (fminunc) [61], which was implemented in the global optimization toolbox of Matlab. B_1_^+^ shimming was applied with three objectives: (i) maximizing minimum B_1_^+^ inside the full ROI; (ii) minimizing the standard deviation (SD) of B_1_^+^ divided by the mean value defining the Coefficient of Variation of B_1_^+^ (CoV = SD/mean); and (iii) maximizing mean B_1_^+^ in the brain ROI [62]. For this purpose, two parallel transmission regimes were used including (i) phase B_1_^+^ shimming (PS) and (ii) amplitude and phase B_1_^+^ shimming (APS) [54].

#### 2.4.2. RF Excitation Vector Optimization for RF Heating and Hyperthermia Treatment Planning

Optimization algorithms are employed to determine optimal excitation vectors for RF coil arrays, ensuring precise and safe heating [63]. For thermal intervention, RF phase and amplitude configurations were determined (independent from the RF phase and amplitude setting used for MRI) by the product of the eigenvector and the square root of its eigenvalue. For excitation vector optimization time multiplexed vector field shaping (MVFS) was implemented [64]. MVFS automatically selects the appropriate time-interleaved excitations. The resulting SAR distribution of the incident electric field interference is tailored to focus the heating of the TR while minimizing local peak RF exposure to healthy and remote tissue below a defined threshold. Finding appropriate constraints to reach temperature target values is essential in this work to reach HT temperature target values in the TR. There is no exact SAR value to reach the HT temperature since the temperature is dependent on many factors in human tissue. Based on the Pennes bioheat equation and using the simplified heat equation SAR = CΔT/Δt (C is the heat capacity in J/°C/kg, cbrain = 3452 J/°C/kg) as an approximation, a temperature increase of 1 °C in 1 min in brain tissue requires a minimum average SAR_10g_ of ~60 W/kg [55]. Following this consideration, SAR_10g_ was set to 40 W/kg < SAR_10g_ < 80 W/kg for the TR to ensure that the target temperature is accomplished in most of the TR. This SAR_10g_ range can be defined as a 0.7 °C < ΔT < 1.4 °C temperature increase in 1 min. For healthy tissues, SAR_10g_ was conservatively limited to <40 W/kg. A safety margin was defined for which SAR_10g_ was not constrained.

### 2.5. Evaluation and Benchmarking

#### 2.5.1. MRI

To assess the performance of the RF applicators for MRI, the transmit performance was evaluated for the phantom and for the voxel model of the human head [65,66].

**B_1_^+^ superposition:**

The evaluation of RF array transmit efficiency necessitates the use of B_1_^+^ amplitudes, as well as the power correlation matrix for each RF channel [65]. For the B_1_^+^ superposition approach, two models accounting for (i) only sample losses (ideal model, IM), and for (ii) sample, coil, and coupling losses (realistic model, RM) were investigated and rescaled to match a total incident power of 1 W (B_1_^+^/√1 W (B_1_^+^_,eff_ [µT/√W]).

**Field shaping for static parallel transmission (pTX):**

The B_1_^+^ maps obtained from the optimization process for B_1_^+^ were rescaled to match a total incident power of 1 kW, which is denoted as B_1_^+^ efficiency (B_1_^+^/√1 kW (B_1_^+^_,eff_ [µT/√kW]), where B_1_^+^ efficiency represents the ratio of B_1_^+^ field strength to the square root of 1 kW.

- Minimum B_1_^+^ optimization:

In order to prevent B_1_^+^ signal dropouts, the objective was to maximize the lowest value of the combined B_1_^+^ field from each individual RF channel over the ROI that encompasses the entire test object or the brain. This was achieved by optimizing the target function of minimum B_1_^+^ [67].

(3) $$\mathrm{Max}\Phi_{\mathrm{TR}}\left({ExH}_{ch}\right)=min\left({\left|\sum_{ch=1}^{N_{ch}}\;B_{1ch}^{+}\cdot {Ex}_{ch}\right|}_{ROI}\right)$$

where *N_ch_* represents the count of channels, $B_{1ch}^{+}$ represents the complex transmission field unique to each RF channel within the 3D ROI, and *Ex_ch_* denotes the complex excitation vector used for *N_ch_* channels [67].

- Coefficient of variation optimization:

The coefficient of variation reflects the degree of (non)uniformity of the B_1_^+^ distribution. In order to reduce the coefficient of variation (CoV, calculated as the standard deviation divided by the mean) over the entire 3D ROI that encompasses the test object or the brain, the following target function was employed [67]:

(4) $$\mathrm{Min}\Phi_{\mathrm{TR}}\left({Ex}_{ch}\right)=\left(\frac{SD\left({\left|\sum_{ch=1}^{N_{ch}}\;B_{1ch}^{+}\cdot {Ex}_{ch}\right|}_{ROI}\right)}{\mathrm{mean}\left({\left|\sum_{ch=1}^{N_{ch}}\;B_{1ch}^{+}\cdot {Ex}_{ch}\right|}_{ROI}\right)}\right)$$

- Mean B_1_^+^ optimization

In order to increase the signal, the objective was to maximize the mean value of the combined B_1_^+^ field from each individual RF channel over the ROI that encompasses the entire test object or the brain. This was achieved by optimizing the target function of minimum B_1_^+^ [67].

(5) $$\mathrm{Max}\Phi_{\mathrm{TR}}\left({ExH}_{ch}\right)=mean\left({\left|\sum_{ch=1}^{N_{ch}}\;B_{1ch}^{+}\cdot {Ex}_{ch}\right|}_{ROI}\right)$$

**SAR optimization with MRI considerations:**

SAR_10g_ is another metric for MRI assessment which should stay within the safety limits for the head (3.2 W/kg for 6 min duration) governed by the IEC guidelines [68]. To assess MR safety the metric SAR_10g,WCS_ (SAR_10g,_ Worst Case Scenario [69]) was used. To obtain SAR_10g,WCS_ maximum |E|^2^ was determined for all possible B_1_^+^ input complex values for each pixel obtained for the APS and PS parallel transmission (pTX) B_1_^+^ shimming approaches [69].

#### 2.5.2. Quality of Targeted RF Heating

For the assessment of the targeted RF, heating SAR-based metrics defined by the ESHO were applied [54]. For further evaluation, we introduced new SAR_10g_-based homogeneity indicators for healthy brain tissues. The input power was scaled until reaching a maximum SAR_10g_ limit of 40 W/kg in healthy tissue. Table 1 surveys the definition and a brief description of all metrics included in the evaluation of the quality of targeted RF heating of the two RF applicator configurations. To examine the quality of the RF heating, we used TC_xx_ as a uniformity factor, which describes the volume that covers xx% (xx = 25, 50, and 75) of the SAR_10g,max_ inside the TR.

#### 2.5.3. Multi-Target Evaluation

**Figure 3:**
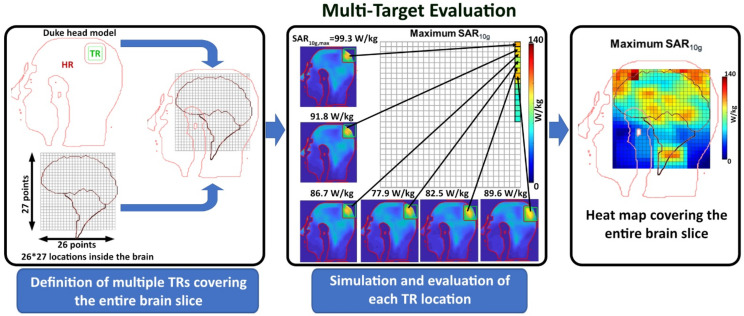
Basic scheme of the multi-target evaluation (MTE) framework. In this approach, hypothetical TRs (borderlines marked in black and green) are moved across the entire brain slice for the evaluation of the quality of targeted RF heating instead of using the conventional approach of defining a very limited number of specific but arbitrary TRs. Simulations were performed for each TR position across a 26 × 27 rectangular grid with 702 points (step size = 7 mm). For evaluation, the metric success score was used, which represents the percentage of the number of locations with SAR_10g,max_ > 40 W/kg over the total number of locations.

Assessment of RF building blocks and ThermalMR phased array applicators commonly uses target region locations covering a few very specific but arbitrary target regions of the brain. Tissue properties inside the human brain exhibit spatial differences, so TRs including tissue with higher conductivity (for example, CSF) can lead to higher SAR_10g_. Following this conventional approach, we placed TR_3_ = 37.5 mm × 37.5 mm × 4 mm (10 mm)) into four arbitrary but specific target regions.

(location L_1_–L_4_) placed in the (i) limbic lobe and postcentral gyrus (L_1_), (ii) the Thalamus region (L2), (iii) the corpus callosum and limbic lobe (L3), and (iv) the parietal lobe (L4) of the brain. Remote, healthy brain tissue was also included in the assessment. However, using a limited number of arbitrary tumor locations in the brain does not represent the full clinical picture. To address this shortcoming, we expanded the evaluation from a limited number of arbitrary tumor locations to whole brain slice coverage. For this reason, we introduced a multi-target evaluation (MTE) framework (Figure 3). For this purpose, numerical simulations moving hypothetical TRs throughout the entire brain (Figure 3, Supplementary Video S1) were conducted (step size = 7 mm, total number of locations = 26 × 27 = 702) for 4 TR sizes: TR_1_ = 30.625 cm^3^ (22 mm) (main target region (cm^3^), gap difference between target margin and safety margin (mm)); TR_2_ = 15.62 cm^3^ (10 mm); TR_3_ = 5.62 cm^3^ (10 mm); and TR_4_ = 0.9 cm^3^ (10 mm). For evaluation, the metric success score was used, which represents the percentage of the number of locations with SAR_10g,max_ > 40 W/kg over the total number of locations.

(15) $$Success\;Score=\frac{Number\;of\;locations\;({SAR}_{10g,max}>40\;W/kg)}{total\;number\;of\;locations}\times 100$$

### 2.6. Data Analysis and Statistics

EMF simulation results including the SAR cuboids derived for each TX channel (matrix including averaged SAR_10g_ for each voxel) obtained from CST were imported into Matlab. For post-processing, the first co-simulations were accomplished. By combining results with co-simulation results and obtaining mesh data, B_1_^+^ superposition, PTX, and averaged SAR_10g_ were calculated. The calculated averaged SAR of each TX channel was determined using quadratic E-fields, imported into Matlab, and used for MVFS optimization. RF-induced heating metrics for thermal therapy were contoured. For data visualization of the RF heating quality metrics, two groups of radar diagrams involving SAR_10g,max_ and more uniform SAR_10g_ in target and healthy tissues were used. The results obtained from the MTE were translated into heatmaps illustrating five metrics including maximum and mean SAR_10g_, and TCxx (%). For the heatmaps, each pixel represents the corresponding metric for each TR location center to compare the performance of the RF applicators. Maps including maximum and mean SAR_10g_ were used to examine the performance of the RF applicators to reach a higher temperature. TCxx intensity maps were used to compare the heat uniformity performance of the RF applicators across the brain. Obtaining two sets of intensity maps helps to identify the applicator that best heats different brain regions with higher uniformity or to find a trade-off between these two features. Statistical analysis of the MTE data obtained for all TRs for each RF applicator was performed using a *t*-test, where *p* ≤ 0.05 was considered to be statistically significant. To examine the quality of the RF heating, we used TC_xx_ as a uniformity factor, which describes the volume that covers xx% (xx = 25, 50, and 75) of the SAR_10g,max_ inside the TR.

## 3. Results

### 3.1. RF Characteristics of the RF Applicators

**Figure 4:**
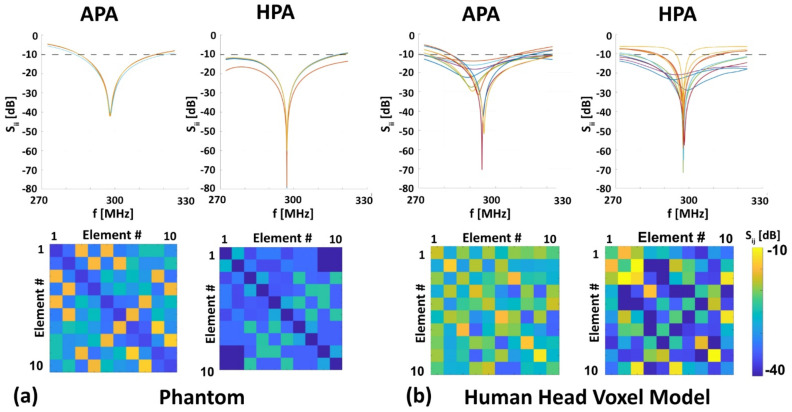
Simulated return loss in 10 different color lines (Sii, top row) and mutual coupling (Sij, bottom row) for the 10-element RF applicators after tuning and matching on (**a**) a phantom and (**b**) a human head voxel model at 297.2 MHz. The dashed line represents −10 dB.

Both RF applicators were tuned and matched to the resonance frequency of 297.2 MHz (±25 MHz) for the phantom (−45 dB < S_ii_ < −40 dB for the APA and −80 dB < S_ii_ < −70 dB for the HPA, Figure 4a, top row) and for the human head voxel model Duke (−70 dB < S_ii_ < −15 dB for the APA and −70 dB < S_ii_ < −20 dB for the HPA, Figure 4b, top row). For the phantom, a bandwidth (Δf|_Sii<−10dB_) of ~30 MHz (APA), and ~48 MHz (HPA) was obtained (Figure 4a). For Duke, a bandwidth of ~35 MHz (APA) and ~4.37 MHz (HPA) was found (Figure 4b). For the phantom mutual coupling, the APA was −25 dB < S_ij_ < −15 dB for any neighboring RF building block. The HPA demonstrated a mutual coupling of −80 dB < S_ij_ < −20 dB (Figure 4a, bottom row). For the Duke, the mutual coupling for the APA was −40 dB < S_ij_ < −15 dB for any neighboring RF building block. The HPA demonstrated a mutual coupling of −70 dB< S_ij_ < −10 dB (Figure 4b, bottom row).

### 3.2. MRI: B_1_^+^ Efficiency, B_1_^+^ Uniformity and RF Power Deposition

**Figure 5:**
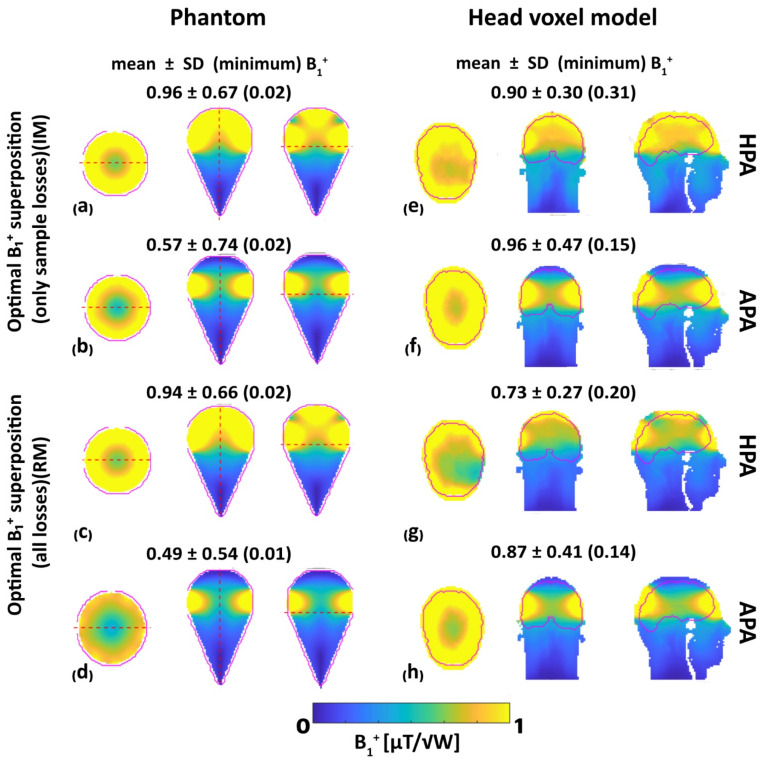
Simulated superposed B_1_^+^ maps for axial, coronal, and sagittal views obtained for the phantom (**left**) and the human head voxel model (**right**). Top (**a,b,e,f**): ideal mode (IM) considering only sample losses, bottom (**c,d,g,h**): realistic mode (RM) including sample, coil, and coupling losses. Annotations highlight mean ± SD (minimum) B_1_^+^ obtained for the HPA and APA RF applicator configurations. The ROI used for the quantitative analysis is highlighted in red.

To examine the benefit of the HPA for MRI, superposed B_1_^+^_,eff_ [µT/√W] distribution maps were determined for the phantom and for the human voxel model Duke. For the phantom, superposed B_1_^+^ efficiency obtained for the HPA in the ROI showed a 67% improvement (IM, mean B_1_^+^_,eff_ = 0.96 µT/√W vs. mean B_1_^+^_,eff_ = 0.57 µT/√W, Figure 5a,b) and a 10% improvement (RM, mean B_1_^+^_,eff_ = 0.94 µT/√W vs. mean B_1_^+^_,eff_ = 0.49 µT/√W, Figure 5c,d) of mean B_1_^+^ over the APA while the minimum B_1_^+^ was similar. For the human head voxel model, B_1_^+^ efficiency obtained for the HPA showed a 6% decrease (IM, mean B_1_^+^_,eff_ = 0.90 µT/√W vs. mean B_1_^+^_,eff_ = 0.96 µT/√W, Figure 5e,f) and 16% decrease (RM, mean B_1_^+^_,eff_ = 0.73 µT/√W vs. mean B_1_^+^_,eff_ = 0.87 µT/√W, Figure 5g,h) in mean B_1_^+^ over the APA. However, the HPA showed a 107% increase (IM, min B_1_^+^_,eff_ = 0.31 µT/√W vs. min B_1_^+^_,eff_ = 0.15 µT/√W, Figure 5e,f) and 43% increase (RM, min B_1_^+^_,eff_ = 0.20 µT/√W vs. min B_1_^+^_,eff_ = 0.14 µT/√W, Figure 5g,h) of minimum B_1_^+^ over the APA for the brain ROI, which reflects its B_1_^+^ efficiency.

Assessment of the B_1_^+^ uniformity across the entire phantom demonstrated a 46% reduction (IM, CoV = 0.7 vs. CoV = 1.3, Figure 5a,b) and a 16% increase (RM, CoV = 0.72 vs. CoV 0.62, Figure 5c,d) in the CoV for the HPA versus the APA. Assessment of the B_1_^+^ uniformity across the entire human brain voxel model demonstrated a 32% reduction (IM, CoV = 0.3 vs. CoV = 0.5, Figure 5e,f) and 20% reduction (RM, CoV = 0.4 vs. CoV = 0.5, Figure 5g,h) CoV for the HPA versus the APA. These results demonstrate an improved B_1_^+^ homogeneity facilitated by the HPA versus the APA.

We examined the MRI metrics derived from PS and APS transmission field shimming of the human head voxel model (Table 2). For the objective of maximizing minimum B_1_^+^, the HPA showed a 4% increase (PS, min B_1_^+^_,eff_ = 2.12 µT/√W vs. min B_1_^+^_,eff_ = 2.04 µT/√W, Table 2) and a 55% increase (APS, min B_1_^+^_,eff_ = 2.71 µT/√W vs. min B_1_^+^_,eff_ = 1.75 µT/√W, Table 2) versus the APA. Transmission field shimming tailored for CoV minimization resulted in similar (PS, CoV = 0.96, vs. CoV = 0.97, Table 2) and a 42% reduction (APS, CoV = 0.51 vs. CoV = 0.88, Table 2) CoV for the HPA versus the APA. Maximizing mean B_1_^+^ revealed a 28% increase (PS, HPA: mean B_1_^+^_,eff_ = 10.6 µT/√W, APA: mean B_1_^+^_,eff_ = 13.61 µT/√W, Table 2) and a 24% increase (APS, HPA: mean B_1_^+^_,eff_ = 11.1 µT/√W, APA: mean B_1_^+^_,eff_ = 13.71 µT/√W, Table 2) provided by the APA versus the HPA.

The outcome of the assessment of the RF power deposition is summarized in Table 3. For the phantom, PS pTx using the HPA (SAR_10g,WCS_ = 2.3 W/kg, Table 3) provided a SAR_10g,WCS_ decrement of 4% versus the APA (SAR_10g,WCS_ = 2.4 W/kg, Table 3).

For APS pTx, the HPA yielded a SAR_10g,WCS_ decrement of 21% versus the APA (HPA: SAR_10g,WCS_ = 14.2 W/kg, APA: SAR_10g,WCS_ = 11.7 W/kg, Table 3. For the human head voxel model, PS pTX using the HPA (SAR_10g,WCS_ = 2.3 W/kg, Table 3) supported a SAR_10g,WCS_ decrement of 15% over the APA counterpart (SAR_10g,WCS_ = 2.7 W/kg, Table 3). SAR_10g,WCS_ obtained for APS pTX using the HPA (SAR_10g,WCS_ = 8.4 W/kg, Table 3) was 11% lower than for the APA (SAR_10g,WCS_ = 9.4 W/kg, Table 3).

### 3.3. RF Heating

#### 3.3.1. SAR-Based Indicators in Four Target Locations

**Figure 6:**
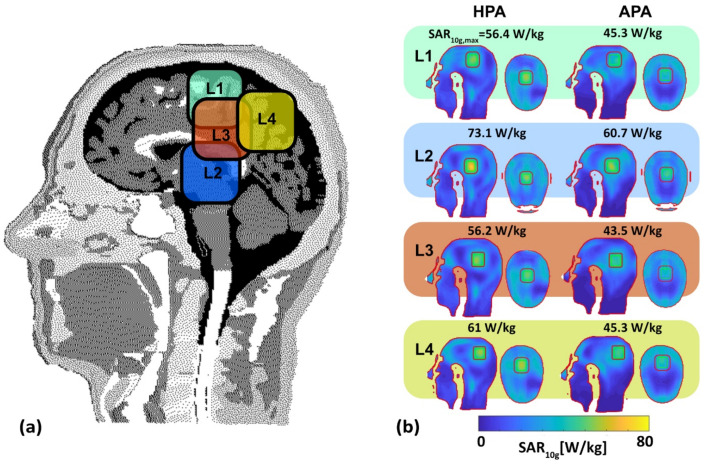
Evaluation of SAR distribution in the human head voxel model Duke. (**a**) Definition of the locations L1–4 of the target regions (TR) placed in the human head voxel model Duke highlighted in four different colors, (**b**) SAR_2q_ maps obtained for the HPA and the APA for the four TR locations (green borderline, volume: 37.5 mm × 37.5 mm × 4 mm = 5.625 cm^3^) placed in the human head voxel model Duke employing MVFS while integrating the total exposure from two to three excitation configurations, which will be executed in a sequentially alternating pattern over time for maximization of SAR_10g_ inside the TR. The size of the safe margin (red borderline) between target and healthy tissues was set to 10 mm. SAR_10g,max_ inside the TR is annotated for the four TR locations for the HPA and APA RF applicator.

Figure 6b shows axial and sagittal SAR_10g_ maps for the four TRs. A quantitative summary of the SAR_10g_-based indicators of the quality of the RF heating is provided in Table 4. 

SAR_10g,max_ (TR) is increased by 25% (L_1_, HPA: SAR_10g,max_ = 56.4 W/kg, APA: SAR_10g,max_ = 45.3 W/kg), 20% (L_2_, HPA: SAR_10g,max_ = 73.1 W/kg, APA: SAR_10g,max_ = 60.7 W/kg), 29% (L_3_, HPA: SAR_10g,max_ = 56.2 W/kg, APA: SAR_10g,max_ = 43.5 W/kg), and 35% (L_4_, HPA: SAR_10g,max_ = 61 W/kg, APA: SAR_10g,max_ = 45.3 W/kg) for the HPA versus the APA. Mean target SAR (MTS) is improved by 30% (L_1_, HPA: MTS = 50 W/kg, APA: MTS = 38.3 W/kg), 18% (L_2_, HPA: MTS = 59.4 W/kg, APA: MTS = 50.2 W/kg), 34% (L_3_, HPA: MTS = 50.4 W/kg, APA: MTS = 37.6 W/kg), and 33% (L_4_, HPA: MTS = 53.4 W/kg, APA: MTS = 40.2 W/kg) for theHPAcompared to the APA. SAR_10g,max_ < 40 W/kg in healthy tissues was achieved for all four locations for both RF applicators which translates to no hotspots. Instead, off-TC_xx_ and THC show the distribution of SAR_10g,max_|_HR_.

**Figure 7:**
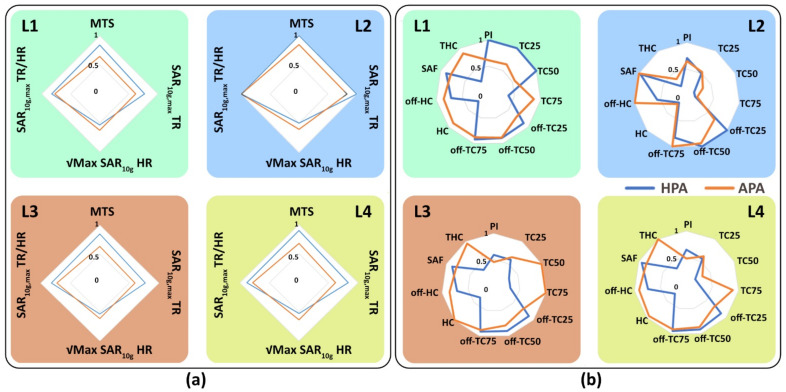
Radar diagrams of the SAR_10g_-based metrics were obtained for the target regions L_1_–L_4_ and for remote, healthy tissue. (**a**) MTS, SAR_10g,max_ in TR, SAR_10g,max_ in HR, and SAR_10g,max_ in TR/SAR_10g,max_ in HR; (**b**) PI, TCxx, off-TCxx, HC, off-HC, SAF, and THC. Results are shown for the HPA (orange lines) and the APA (blue lines). The boxes color-coded based on Figure 6.

For the HPA the SAR_10g_ amplification factor SAF was increased (L_1_: 7%, HPA: SAF = 3.42, APA: SAF = 3.21; L_3_: 12%, HPA: SAF = 3.39, APA: SAF = 3.02; L_4_: 12%, HPA: SAF = 3.65, APA: SAF = 3.26)) for three of the four TR locations while it was similar for L_2_ (HPA: SAF = 4.13, APA: SAF = 34.16). For enhanced visualization of the RF heating results, Figure 7 illustrates the SAR_10g_-based metrics in two groups obtained for the target regions L_1_–L_4_ and for remote, healthy tissue. The performance indicator PI for the HPA was superior to the APA. Table 4 summarizes the RF heating efficiency and uniformity obtained for the HPA and the APA for L_1_–L_4_.

#### 3.3.2. Multi-Target Evaluation

**Figure 8:**
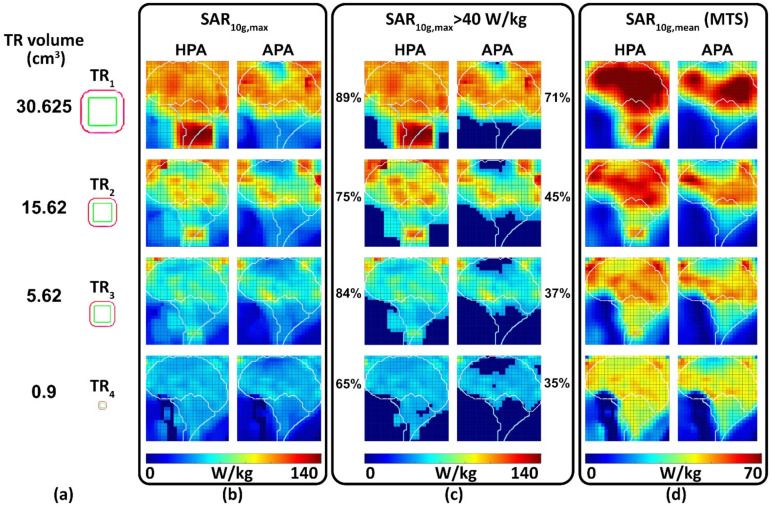
Summary of the results obtained from the multi-target evaluation (MTE) of the human head voxel model Duke. (**a**) Four TR volumes (cm^3^) were used. Maps obtained for (**b**) SAR_10g,max_, (**c**) SAR_10g,max_ > 40 w/kg and annotated by success scores showing the percentage of TR locations (voxels) reaching the acceptable SAR_10g,max_ higher than 40 W/kg divided by the total number of voxels (702), and (**d**) maps derived for mean SAR_10g_ in the target region (MTS). The outline of the brain is depicted in white. Some target positions are outside of the brain but inside the head of the human voxel model.

Figure 8 shows the distributions of SAR_10g,max_, SAR_10g,max_ > 40 W/kg, and MTS, for a central sagittal slice of the brain. The success score (Figure 8c) shows a 25% (HPA: 89%, APA: 71%), 67% (HPA: 75%, APA: 45%), 127% (HPA: 84%, APA: 37%), and 86% (HPA: 65%, APA: 35%) improvement for the HPA versus the APA.

**Figure 9:**
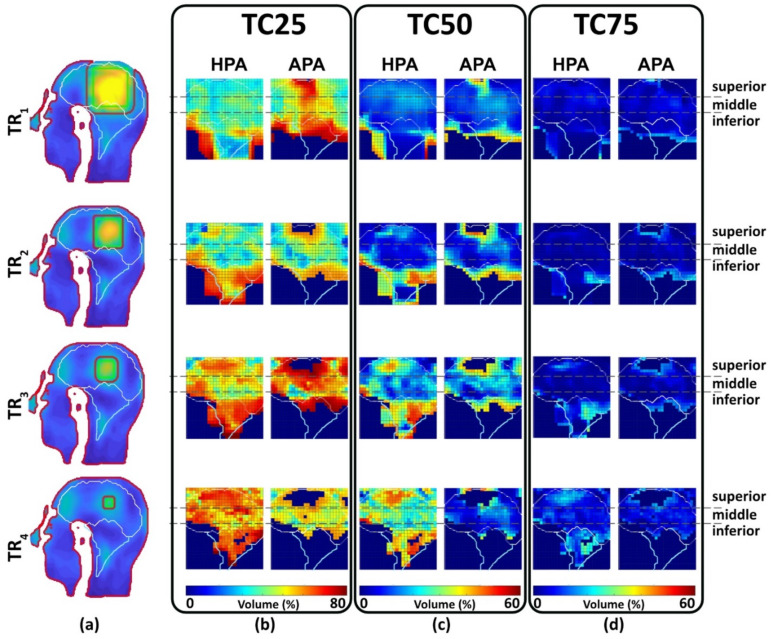
Analysis of SAR_10g_ distribution and target coverage in the brain of the human voxel model Duke. (**a**) SAR_10g_ distribution for four TR sizes using RF focusing for one arbitrary location in the brain. (**b**–**d**) Target coverage (TCxx = percent of volume covered by xx% of SAR_10g,max_ > 40 W/kg in the TR) was obtained for 702 locations across a mid-sagittal slice of the brain for (**b**) TC_25_, (**c**) TC_50_, and (**d**) TC_75_. The gray dash lines highlight the superior, middle, and inferior regions of the brain. Regions with SAR_10g_ < 40 W/kg are marked dark blue.

For the large TR_1_, TC_xx_s provided by the APA were better than the HPA counterparts for the superior regions of the brain (Figure 9). For the mid and deep inferior regions of the brain, the HPA showed higher TCxxs than the APA (Figure 9). For middle brain regions both RF applicators showed similar uniform SAR_10g_ distribution in the TRs. For the smaller TRs TR_2_–TR_4_, TC_xx_s obtained for the HPA were better than the APA for all brain regions included in MTE (Figure 9).

**Figure 10:**
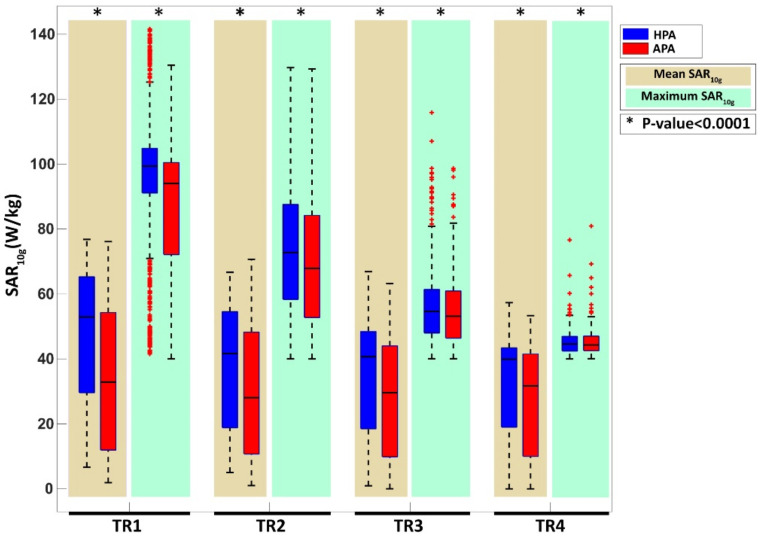
Comparison of the helmet (blue, HPA) and the annular array (red, APA) for all TRs and locations included in the multi-target evaluation. Results obtained for mean SAR_10g_ in the target region (MTS) are marked with beige bars. Results obtained for the SAR_10g,max_ are marked with green bars. (* *p* < 0.0001).

For a quantitative comparison, Figure 10 shows box plots of the SAR_10g,max_ and MTS for all TRs and locations included in the multi-target evaluation of the HPA and the APA. The highest SAR_10g,max_ (142 W/kg) and MTS (128 W/kg) was achieved by the HPA. Statistical analysis showed that the HPA was superior to the APA for all TRs (*p* < 0.0001) although the interquartile range is very close (MTS: 11 W/kg (HPA) and 7 W/kg (APA), SAR_10g,max_: 18 W/kg (HPA), 19 W/Kg (APA)).

#### 3.3.3. Temperature Simulations

**Figure 11:**
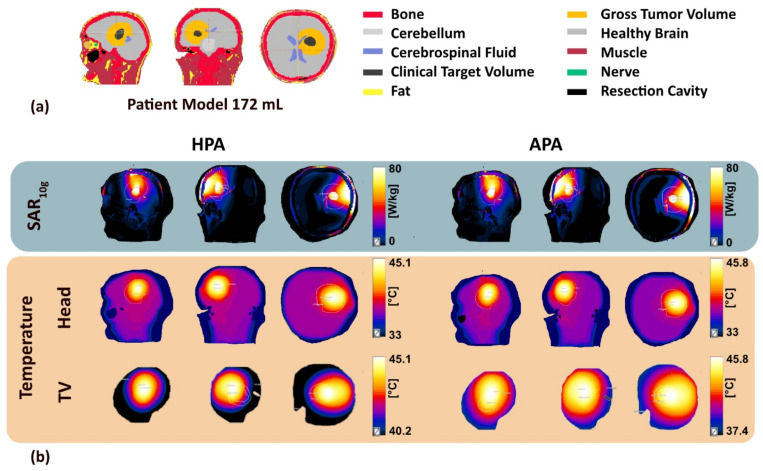
Summary of results obtained for (**a**) a realistic clinical case, in which the human head voxel model was generated with CT data obtained from a patient with glioblastoma multiforme (172 mL, total head mass: 3.68 kg TV, bounding box size: (58, 65, 68) m^3^) using HypT optimizer tool of Sim4Life followed by (**b**) SAR_10g_ (first row), temperature simulations for the head voxel model (second row), and clinical TV (third row) using the HPA (left) and the APA (right).

**Figure 12:**
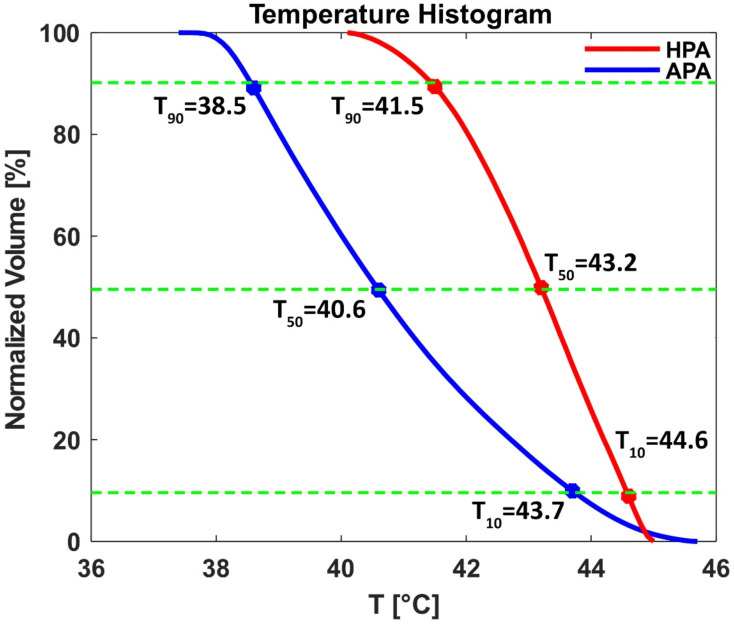
Temperature volume histogram inside the tumor obtained using HypT optimizer tool of Sim4Life for the APA (blue line) and the HPA (red line). The cumulative normalized volume covered by different temperatures is expressed by T_10_, T_50_, and T_90_. The RF heating achieved for the HPA outperformed the APA. Green dotted lines show 10%, 50% and 90% normalization volume.

SAR_10g_ and temperature distributions obtained for a clinical case of glioblastoma multiforme using Sim4Life “HypT optimizer” are illustrated in Figure 11. The temperature simulations yielded T_min_ = 40.2 °C for the HPA and 37.4 °C for the APA inside the TR. For the heating paradigm simulated, a maximum temperature of T_max_ = 45.1 °C was found in the TR for the HPA. For the APA T_max_ = 45.8 °C was observed in the tumor TR. Figure 12 shows a cumulative plot of the temperature distribution in the tumor TR. The RF heating achieved for the HPA outperformed the APA. The HPA provided T_90_ = 41.5 °C, T_50_ = 43.18 °C, and T_10_ = 44.54 °C while the APA provided T_90_ = 38.6 °C, T_50_ = 40.63 °C, and T_10_ = 43.7 °C.

## 4. Discussion

This study demonstrates that helmet RF applicators improve the efficacy of ThermalMR by providing enhanced transmission field uniformity for brain MRI, and by enabling enhanced focal point quality for RF-induced heating of brain tumors compared to an annular RF array using the same number of transmit RF elements. The Helmet RF phased array enhances ThermalMR’s RF heating quality and transmission field uniformity across the brain, not by increasing the number of TX channels, but through an optimized rearrangement, as it was shown that 16 elements can outperform 32 elements [10]. Given the limited surface area of the head and spatial constraints, the HPA strategically places two SGBT elements on top of the head. This innovative configuration overcomes spatial limitations and improves the performance of the RF array by ensuring a more effective and uniform distribution of RF energy, demonstrating a significant advancement in hyperthermia therapy and ThermalMR technologies without the need for increasing element density. Our EMF and temperature simulations confirm the hypothesis that these transmission elements placed on top of the head improve performance criteria critical to MRI and enhance the temperature focusing of conventional annular array RF applicators, especially in the superior regions of the brain.

Published reports on dome-shaped RF applicators customized for targeting brain regions have shown that these transmitter array configurations have value for UHF-MR. However, none of these published RF arrays used transmission elements placed perpendicular to the cranial–caudal axis of the body or to the main magnetic field of an MR scanner [21,31,36,37]. The current study is the first report demonstrating the performance improvement of a helmet RF applicator for ThermalMR over an APA using the same number of transmission elements. This improvement facilitates the integration of diagnostic imaging guidance, thermal treatment, and MR temperature cartography-based monitoring of RF heating in a single, multi-purpose RF applicator to enhance thermal theranostics, and to better describe the role of temperature in biological systems and disease. In [10,21], different types of APAs were compared to find the most advanced solution for APAs. This work builds upon an APA configuration, enhancing it with the HPA by adding two additional elements to an 8-channel APA, thus broadening brain coverage. This improvement recognizes that brain tumors and target areas have varied shapes and sizes, often extending beyond the APA’s typical coverage. Moreover, the RF applicator setup should be centered over the brain to maintain accuracy to support MR imaging and thermometry during treatment. The adaptability of the RF applicator, crucial for effective therapy, is retained in the HPA configuration. It allows for the adjustment of RF elements along the head for optimal positioning relative to the tumor location, provided that B_1_^+^ homogeneity remains acceptable in the Region of Interest (ROI). This highlights the significance of a flexible and movable RF applicator system like the HPA, which not only enhances the traditional APA setup but also ensures optimized treatment and imaging efficiency, regardless of the tumor’s size, shape, or location within the brain. A limitation of our study is the limited number of transmission elements used. The restriction to using only ten SGBT elements for the annular array reference is a result of the minimum inter-element distance and inter-element coupling constraints. The HPA demonstrated an improved mutual decoupling of ~10%. Adding an extra ring of TX elements along the cranial–caudal axis [10] would enhance anatomical coverage in the superior–inferior direction and provide extra degrees of freedom for optimizing excitation vectors tailored for MRI and RF heating. This benefit could be exploited for further SAR_10g_ reduction and B_1_^+^ uniformity improvements for MRI and enhanced targeted RF power deposition in deep-seated brain tumors using annular array configurations. However, adding a second ring to the annular array would double the number of TX channels. This would present a cost constraint, driven primarily by the costs of the RF power amplifiers. This would also pose a practical obstacle since current pTX systems of MRI scanners are limited to 8–16 independent transmission channels. In previous approaches to ThermalMR RF applicator design, increasing the number of elements from 8 to 32 in different numbers of rows was studied [10,21]. In this simulation study, 10-SGBT was the maximum number of TX elements that can fit in one APA to compare its full potential with an APA. Therefore, the HPA approach is more cost-effective than multiple rings of TX elements used in an annular array. Beyond the helmet RF applicator with ten TX elements examined in this proof-of-principle study, the range of possible alternative helmet RF applicator configurations is even larger. Our study demonstrates for the first time that the helmet design approach is promising for MRI and HT, and further explorations of alternative helmet-based applicator configurations are likely to prove fruitful.

The HPA configuration examined in this work takes the elliptical shape of the human head into account and aligns the long axis of the SGBT building blocks placed on top of the head with the longest (superior-inferior) axis of the head for the benefit of better head and brain coverage. Aligning the long axis of the SGBT building blocks placed on top of the head with the left–right axis of the head would also be a viable alternative arrangement. Our simulations used the same distance from the center of the left–right direction of the brain for each SGBT building block placed on top of the head. Asymmetric positioning of the SGBT buildings placed on top of the head around the center of the left-right direction of the brain is an alternative approach. The center point of the SGBT building blocks placed on top of the head was aligned with the center of the anterior-posterior direction of the brain. However, off-center positioning of the SGBT buildings placed on top of the head is also feasible.

The reproducibility of EMF simulations is an ongoing discussion. This is not only due to insufficient disclosure and description of the physical setups. Additionally, there are typically too many parameters to be specified in the simulation process, and it is hardly feasible to repeat or reproduce the entire set of parameters in a study. Finally, these parameters may change with every simulation tool and algorithm, with even every simulation software version and human voxel model. Fortunately, these constraints typically do not compromise the main results of an EMF simulation, but—in a strict sense—can be critical for the important principle of reproducibility. Therefore, in this work, phantom results served as a form of ground truth to be easier reproduced although our main results are obtained using the Duke human head voxel models. Analyzing results with the MTE approach, Figure 8 and Figure 9 reveal extensive details on RF applicator performance. To mimic a realistic clinical scenario, our simulations include patient-specific model extraction (n = 1) obtained from CT images. For clinical application details about the type of tumor tissue (extremely dense, heterogeneously dense, scattered fibroglandular and predominantly fatty, non-uniform perfusion) need to be included in the hyperthermia treatment planning [74].

Figure 8 shows APA’s inconsistent performance versus HPA in superior regions for TR1, with a decline to zero in TR2 and TR3, highlighting the top antennas’ importance. The reason behind the superior performance of the HPA over the APA in the lower brain/spinal cord area in Figure 8 for TR1 might be because of EM wave interference of top antennas inside the MR waveguide [75].

Figure 9 introduces TCxx, differing from maximum or mean SAR, as a homogeneity measure of SAR distribution within TRs, affected by SAR range. The MTE approach illustrates a reduction in TCxx, influenced by TR dimensions and the diverse sizes and thermal properties of brain tissues, complicating TCxx predictions. The Supplementary Videos S1–S9 illustrate SAR distribution variances across TRs and locations. This complexity emphasizes the MTE’s role in dissecting these relationships. Figure 9 shows TCxx values decreasing from TC25 to TC75 across TRs, with TC75 nearing zero for all, indicating minimal variance. Our MTE approach provides a technical foundation for a more objective RF applicator evaluation using whole-brain accessibility instead of a limited set of specific but arbitrary target locations, which may not represent the whole clinically relevant picture. This achievement presents a foundation for further RF applicator design tailored for ThermalMR-based therapy of brain tumors. The MTE approach has the potential to become a standard for benchmarking RF-applicator designs for a broad range of applications, not only limited to the brain but also for HT in other anatomical targets including extremities and abdomen. Timely computation and optimization of the excitation vectors targeting the high number of TR locations used for MTE is facilitated by the time multiplexing vector field shaping approach. The MVFS approach efficiently handles extensive sets of optimization constraints and provides an optimal solution through a minimal number of rapid iterative computations. The runtime is only minimally influenced by the total quantity of SAR matrices within the model. A computation time for each time-interleaved solution <1 s was used for an individual target using a mid-tier consumer PC. Considering 2–3 time intervals for each TR, whole brain slice coverage required a computation time of ~14 h. These computation times can be reduced using parallel computing. Further acceleration can be achieved by spatial undersampling to reduce the number of target points with respect to the TR size. With the MVFS, excitation vectors can be customized to match the size and arbitrary geometry of individual tumor volumes to meet the HT planning requirements of individual patients and targets. In this work, we used cuboidal target regions rather than cylindrical or spherical TRs to make the TR more unpredictable, and less symmetrical, than the symmetrical applicator arrangement of the annular array. This cuboidal target poses greater challenges for RF targeting compared to spherical TRs, primarily due to the typical development of beamforming techniques focused on the center of the target region. MVFS-based excitation vector optimization is not limited to a single CTV or GTV but also supports simultaneous targeting of multiple CTVs or GTVs [44]. This is consistent with the needs of personalized medicine and can be achieved without necessitating patient-specific RF applicator hardware. The performance improvement using HPA is not dependent on the focusing algorithm but it is related to the antenna arrangement. The HperT optimizer tool in Sim4life was utilized for this purpose.

It is a recognized limitation of our proof-of-principle study that the traditional Pennes Bioheat equation has been used for temperature simulations. This approach uses an approximation of uniform or isotropic blood perfusion. Based on the ESHO benchmarks for computational modeling and optimization in hyperthermia therapy [54], our work used blood perfusion rates (kg/s/m^3^) for muscle, fat, and tumor to approximate heterogeneities in the blood perfusion under baseline and under thermal stress. Very recently, an advanced version of the Pennes bioheat equation was proposed to take into account heterogeneous or anisotropic blood perfusion [76].

Replacing the water bolus filled with deionized water placed between the RF building blocks and the head with high dielectric materials offers another research direction to further improve the MRI performance and heating efficacy. The use of high permittivity materials would enhance wavelength shortening and would facilitate size reduction of the SGBT building blocks [77]. The preference would be high permittivity slurries over ceramics to maintain some of the cooling effects of the water bolus. The use of metamaterial surfaces is also conceptually appealing for pursuing the development of ThermalMR applicators due to the extra degrees of freedom for shaping electromagnetic wave propagation and constructive interference in the target region [77,78,79]. This benefit could facilitate SAR_10g_ reduction and B_1_^+^ uniformity improvements for MRI and enhance targeted RF power deposition in deep-seated brain tumors.

## 5. Conclusions

This work demonstrates the enhanced performance of the helmet RF applicator compared to an annular array in terms of transmission field coverage and uniformity across the brain, which is essential for MRI diagnostics of cancer. Our findings document that the HPA facilitates a ~10% improvement of SAR_10g,max_ in the target region versus the annular array, which augments hyperthermia treatment as an adjunct to chemo- and radiotherapy of brain tumors. To conclude, our results provide a technical foundation for objective RF applicator evaluation using whole brain slice coverage instead of a few specific but arbitrary target locations using the MTE approach. Our findings constitute a foundation for the experimental implementation and application of a helmet array driven by broadband self-grounded bow tie antenna building blocks en route to ThermalMR theranostics of brain tumors.

## Figures and Tables

**Table 1:** SAR_10g_-based metrics used for the evaluation of the quality of the targeted RF heating.

Indicator	Location	Description	Unit	Valid Value	Equation
**SAR_10g_**	Whole Head	SAR_10g_ averaging over cubes covering 10 g of tissue (SAR_10g_) distributions inside the human head voxel model Duke $SAR=\frac{\sigma {\left|E\right|}^{2}}{2\rho }$; σ (S/m) is the electrical conductivity, ρ (kg/cm^3^) is the mass density, and |E| (V/m) is the magnitude of the local electric field vector	W/kg	<40 (HR)	(6)
				<80 >40 (TR)	
**TCxx (TC_25_/TC_50_/TC_75_)** [70,71,72]	TR	Measures the percentage of volume with SAR_10g_ over 25% (TC25), 50% (TC50) or 75% (TC75) of SAR_10g,max_ found in the TR. This is only evaluated if SAR_10g,max_ >40 W/kg. $TC_{xx}=\frac{voxel_{TR}{|}_{{{SAR}_{10g}>xx.SAR}_{10g,max}}}{totalvoxel_{TR}},xx=25,50,75$	%	>75 [54]	(7)
* Off-TCxx (off-TC_25_/off-TC_50_/off-TC_75_)	HR	iso-SAR contour in healthy tissue defined as off-target regions (off-TR). Measures the percentage of voxels in the HR with SAR_10g_ over the (25%, 50%, and 70%) of the SAR_10g,max_ found in HR (healthy constraint SAR_10g_ (40 W/kg))). $off-TC_{xx}=\frac{voxel_{HR}{|}_{{{SAR}_{10g}>xx.SAR}_{10g,max}}}{totalvoxel_{HR}},xx=25,50,75$	%		(8)
**SAR_10g_ Amplification Factor (SAF)** [10,70,73]	Whole Head	Measures that healthy tissue preservation from mean SAR_10g_ in HR $SAF={SAR}_{10g,mean}\left(TR\right)/{SAR}_{10g,mean}\left(healthy\right)$	-		(9)
**Homogeneity Coefficient (HC)** [70]	TR	Measures how homogenous the SAR_10g,max_ is distributed over TR	-		
		HC(TR)=TC(75)/TC(25)	-		(10)
* Homogeneity Coefficient (off-HC)	HR	Measures how homogenous the SAR_10g,max_ in healthy tissue is distributed over HV.	-		
		off−HC(HVR)=(off−TC(75))/(off−TC(25))	-		(11)
* Total Homogeneity **Coefficient (THC)**	Whole Head	Measures total homogeneity of SAR_10g_ inside the TR and HR to satisfy hyperthermia treatment goals	-		
		HC(TR)=HC.off−HC	-		(12)
**Performance Indicator (PI)** [72]	Whole Head	Measures the total performance of each hyperthermia treatment planning	-		
		$PI=SAR_{\left(10g,max\right)}{|}_{TR}\;.SAF.TC$	W/kg		(13)
**T_xx_ (T_10_/T_50_/T_90_)** [54,72]	TR	In clinical practice, the assessment of tumor coverage should involve the examination of indexed temperatures, specifically T10, T50, and T90. These values correspond to the temperatures reached in at least 10%, 50%, and 90% of the target region, respectively.	°C	T_50_ > 40	(14)

* These indicators are defined for this work.

**Table 2:** Summary of the MRI performance of the RF applicators using the objective of maximization of minimum B_1_^+^ (Equation (2)), minimization of the coefficient of variation (CoV, Equation (3)), and maximization of mean B_1_^+^ across the brain of the human head voxel model Duke. Green and red numbers highlight the results obtained for each optimization goal outlined in the very left column. Gray numbers review results obtained outside of the optimization goal outlined in the very left column.

Optimization Goal	pTx Shimming Method	Min B_1_^+^ (µT/√kW)		Coefficient of Variation		Mean B_1_^+^ (µT/√kW)	
		Helmet Array	Annular Array	Helmet Array	Annular Array	Helmet Array	Annular Array
Max (min B_1_^+^)	PS	2.12	2.04	0.98	1.41	8.82	9.89
	APS	2.71	1.75	2.87	1.62	8.78	10.81
min (CoV)	PS	0.06	0.48	0.96	0.97	8.10	11.01
	APS	0.02	1.62	0.51	0.88	3.68	11.47
Max (Mean B_1_^+^)	PS	0.047	0.087	0.95	1.12	10.6	13.61
	APS	0.34	0.01	1.33	1.99	11.1	13.71

**Table 3:** Worst case scenario SAR_10g_ (SAR_10g,WCS_), obtained for the phantom and for the human head voxel model Duke using amplitude and phase (APS) or phase only (PS) parallel transmission field shimming for the helmet (HPA) and for the annular (APA) RF applicator.

		SAR_10g,wc_ (W/kg)	
pTx Shimming Method	RF Applicator	Phantom	Human Head Voxel Model
PS	HPA	2.3	2.3
	APA	2.4	2.7
APS	HPA	11.7	8.4
	APA	14.2	9.4

**Table 4:** Assessment of RF heating efficiency and uniformity. SAR-based metrics defined in Table 1 were examined for four target regions L1–L4 (Figure 6a) placed in the human head voxel model Duke. Remote, healthy brain tissue was included in the assessment. The table is color-codded based on Figure 6.

	L1		L2		L3		L4	
Locations	Limbic Lobe and Postcentral Gyrus		Thalamus		Corpus Callosum and Limbic Lobe		Parietal Lobe of the Brain	
Metrics								
	HPA	APA	HPA	APA	HPA	APA	HPA	APA
**MTS (W/kg)**	49.9	38.3	59.4	50.2	50.4	37.6	53.4	40.2
**Max SAR_10g_ TR (W/kg)**	56.4	45.3	73.1	60.7	56.2	43.5	61.0	45.3
**Max SAR_10g_ HR (W/kg)**	16.5	14.1	17.7	14.6	16.6	14.4	16.7	13.9
**SAF**	3.42	3.21	4.13	4.16	3.39	3.02	3.65	3.26
**Max SAR_10g_ TR/HR (%)**	0.23	0.19	0.16	0.17	0.25	0.23	0.25	0.259
**TC_25_ (%)**	88.5	56.3	44.1	47.0	52.1	57.1	49.5	54.2
**TC_50_ (%)**	52.7	29.0	7.8	17.4	19.4	31.3	16.3	29.3
**TC_75_ (%)**	4.5	10.2	1.8	2.3	3.7	11.7	3.0	10.5
**HC**	0.05	0.18	0.04	0.05	0.071	0.21	0.060	0.19
**Off-TC_25_ (%)**	73.2	58.2	82.6	56.6	73.1	58.7	72.5	56.9
**Off-TC_50_ (%)**	34.9	34.6	38.9	36.5	35.2	35.3	35.7	33.8
**Off- TC_75_ (%)**	16.4	15.7	14.8	17.8	16.4	15.8	16.9	16.3
**Off-HC**	0.22	0.27	0.18	0.31	0.22	0.27	0.233	0.29
**THC**	0.01	0.05	0.01	0.02	0.01	0.06	0.01	0.06
**PI**	149.7	68.9	106.4	97	87.9	67.1	96.6	71.2

## Data Availability

The data presented in this study are available on request from the corresponding author due to privacy reasons.

## Supplementary Materials

The following supporting information can be downloaded at https://www.mdpi.com/article/10.3390/bioengineering11070733/s1. Video S1: MTE. The basic scheme of the multi-target evaluation (MTE) framework is shown in this video for a specific TR_3_ size, using the HPA for mapping SAR_10g,max_. For MTE, hypothetical TRs (borderlines marked in black and green) are moved across the entire brain slice for the evaluation of the quality of targeted RF heating instead of using the conventional approach of defining a limited number of specific but arbitrary TRs. EMF simulations were performed for each TR position across a 26 × 27 rectangular grid with 702 points (step size = 7 mm). For evaluation, the metric success score was used, which represents the percentage of the number of locations with SAR_10g,max_ > 40 W/kg over the total number of locations. Videos S2–S9: APA/HPA_TR1-TR4_MTE.
